# Impact of M‐protein detection on the response evaluations of patients undergoing treatment with the IgG‐κ monoclonal antibodies daratumumab or isatuximab, and discrepancies between immunofixation electrophoresis (IFE) systems and reagents

**DOI:** 10.1002/cam4.70128

**Published:** 2024-08-23

**Authors:** Yuko Shirouchi, Kazumi Kaihara, Tsunaki Sekita, Naoko Amano, Konosuke Nakayama, Kazunori Miyake, Hitoshi Abe, Hirotoshi Oinuma, Dai Maruyama

**Affiliations:** ^1^ Department of Hematology Oncology Cancer Institute Hospital, Japanese Foundation for Cancer Research Tokyo Japan; ^2^ Department of Clinical Laboratory Cancer Institute Hospital, Japanese Foundation for Cancer Research Tokyo Japan; ^3^ Scientific Affairs Sebia Japan; ^4^ Application Sebia Japan

**Keywords:** cancer management, chemotherapy, hematological cancer, multiple myeloma

## Abstract

**Background:**

Immunofixation electrophoresis (IFE) is the standard method for confirming the presence of a monoclonal protein (M‐protein) at multiple myeloma (MM) diagnosis. IFE is also essential at assessment of complete response (CR) and stringent CR during treatment. As the CR assessment is influenced by daratumumab and isatuximab, HYDRASHIFT assays were developed.

**Methods:**

Samples from patients under treatment that included daratumumab or isatuximab were tested and monitored by IFE on the HYDRASYS system using HYDRASHIFT assays (HYDRASYS/HYDRASHIFT) and by IFE on the Epalyzer2 system (Epalyzer).

**Results:**

The IFE using HYDRASYS/HYDRASHIFT avoided a false positive caused by drug‐related IgG‐κ and contributed to accurate assessment of CR. Furthermore, HYDRASYS/HYDRASHIFT detected small M‐proteins at early relapse and detected free light chains (FLCs) in patients with renal impairment exhibiting high serum FLCs despite being often missed on Epalyzer.

**Conclusion:**

Sensitivity and specificity of M‐protein detection vary greatly depending on the IFE system and reagents used.

## 1 INTRODUCTION

Immunofixation electrophoresis (IFE) is utilized for M‐protein detection and is required for assessing complete response (CR) and stringent complete response (sCR) in the treatment of MM, according to the IMWG uniform response criteria,^1^ and the determination of CR should not be affected by unrelated M‐proteins that are secondary to therapeutically administered monoclonal antibodies.^2^ Daratumumab and lsatuximab are anti‐CD38 monoclonal antibodies (mAb) that improve outcomes and depth of response in multiple myeloma (MM) patients. Both daratumumab and isatuximab are IgG‐κ mAb detected by IFE, interfering with interpretation of CR and sCR. To avoid this interference on the serum IFE and assist in accurate clinical assessment and therapeutic monitoring of MM patients, the HYDRASHIFT 2/4 daratumumab (daratumumab‐specific immunofixation reflex assay, DIRA)^3^, ^4^ was developed by Sebia with Janssen, and additionally, the HYDRASHIFT 2/4 isatuximab^5^ was developed by Sebia with Sanofi. These assays have been used in determining final CR and sCR in clinical studies^6^, ^7^, ^8^ and in patients under anti‐CD38 mAb treatment in clinical practices.^9^, ^10^, ^11^

## 2 METHOD

The HYDRASHIFT assays for daratumumab (HYDRASHIFT Dara IF assay) and isatuximab (HYDRASHIFT Isa IF assay), intended for use on the HYDRASYS system (Sebia, Lisses Evry Cedex, France), were evaluated for providing accurate M‐protein detection and CR assessment in MM and compared to results from another IFE system, the Epalyzer2 (Helena, TX, USA).

Samples from patients whose treatments included daratumumab were tested by IFE with the HYDRASHIFT Dara IF assay on the HYDRASYS2 system (Sebia) and with the Epalyzer2. Samples from patients whose treatments included isatuximab were tested and monitored by IFE with the HYDRASHIFT Isa IF assay on the HYDRASYS2 system, and with the Epalyzer2. Serum free light chains (FLCs) and immunoglobulin (IgG, IgA, IgM) concentrations were also measured.

## 3 RESULTS

### 3.1 Patient characteristic at the time of response assessment

The median age of 12 patients under daratumumab (7 males and 5 females) was 72 years (range: 57–80) and the median age of the patients under isatuximab (3 males and 2 females) was 73 years (range: 49–79). As shown in Table 1, the HYDRASHIFT Dara IF assay completely removed the daratumumab‐based IgG‐κ band that was detected by Epalyzer (IgG‐κ italicized in Table 1) in the gamma region, changing 2 of the 12 patients (16.7%) to a sCR response evaluation (patients 8 and 9 in Table 1). The other 10 patients showed the MM‐originating endogenous M‐proteins, IgG‐κ, IgG‐λ, IgA‐κ, and Free‐κ. The comigrating daratumumab/IgG‐κ bands were present in 5 of the 12 patients (patients 2, 3, 4, 5, and 6 in Table 1), or 38.5%. This result resembles a previous report where the HYDRASHIFT Dara IF assay was performed at the Memorial Sloan Kettering Cancer Center on 96 patients, 43 (44.8%) of which had comigrating daratumumab/IgG‐κ IF bands.^8^

**TABLE 1:** Patient characteristics at the time of response assessment under daratumumab and isatuximab treatment.

Patient	Age/Sex	MM subtype	Treatment	Creatinine clearance^a^ (mL/min)	Detected type of M‐protein		serum IGs (mg/dL)			serum FLCs (mg/L)			Response evaluation by HYDRASHIFT	Response evaluation by Epalyzer
					HYDRASHIFT/HYDRASYS	Epalyzer	IgG	IgA	IgM	κ	λ	κ/λ		
1	79/M	IgG‐λ	Dara	47.3	IgG‐λ + free‐κ	IgG‐λ, *IgG‐κ* ^b^	563	3	9	<0.5	8.8	<0.06	PR	PR
2	57/M	IgG‐κ	Dara	88.4	IgG‐κ	IgG‐κ	702	34	9	15.8	1.5	10.53	PR	PR
3	69/M	IgG‐κ	Dara	75.5	IgG‐κ	IgG‐κ	302	7	4	<0.5	0.9	<0.56	PR	PR
4	74/F	IgG‐κ	Dara	70.3	IgG‐κ	IgG‐κ	1291	27	20	161	7.7	21	PD	PD
5	71/M	IgG‐κ	Dara	49.9	IgG‐κ	IgG‐κ	1079	26	8	42.1	2.6	16.19	PR	PR
6	71/F	IgG‐κ, Free‐κ	Dara	50.4	IgG‐κ	IgG‐κ, *IgG‐κ* ^b^	670	19	19	11.8	6.6	1.79	PR	PR
7	77/M	IgG‐λ, Free‐λ	Dara	79.9	IgG‐λ	IgG‐λ, *IgG‐κ* ^b^	1748	21	11	0.6	12.4	0.05	PR	PR
8	73/F	Free‐κ	Dara	46.4	Not detected	*IgG‐κ* ^b^	1083	361	31	18	17.5	1.03	sCR	VGPR
9	73/F	IgG‐κ, Free‐κ	Dara	49.4	Not detected	*IgG‐κ* ^b^	592	50	13	7	7.8	0.9	sCR	VGPR
10	71/M	IgG‐λ	Dara	78.2	IgG‐λ	IgG‐λ, *IgG‐κ* ^b^	557	35	28	5	10.1	0.5	VGPR	VGPR
11	69/M	IgA‐λ	Dara	51.3	IgA‐κ	IgA‐alone^c^, *IgG‐κ* ^b^	415	71	8	6.3	12.5	0.5	VGPR	VGPR
12	80/F	IgA‐κ	Dara	80	IgA‐κx2	IgA‐κ, *IgG‐κ* ^b^	349	909	12	5.7	0.9	6.33	SD	SD
13	71/F	IgG‐λ	Isa	56.2	Not detected	*IgG‐κ* ^b^	276	11	3	< 0.5	1.7	< 0.29	CR	CR
14	58/M	IgG‐κ	Isa	46.6	IgG‐κ	IgG‐κ	2473	4	3	4.1	0.5	8.2	PD	PD
15	73/M	Free‐κ	Isa	21.2	IgA‐κ + free‐κx2	IgA‐alone^c^, Free‐κ, *IgG‐κ* ^b^	269	48	9	1585	6.9	229.7	PD	PD
16	79/F	IgA‐κ, Free‐κ	Isa	27.8	Not detected	*IgG‐κ* ^b^	122	8	2	< 0.5	0.8	< 0.63	CR	CR
17	49/M	IgG‐λ	Isa	84.5	IgG‐λ	IgG‐λ, *IgG‐κ* ^b^	362	17	7	< 0.5	1.3	< 0.38	VGPR	VGPR

Abbreviations: CR, complete response; Dara, daratumumab; Epalyzer, IFE assay on the Epalyzer2 system (Helena); F, female; HYDRASHIFT/HYDRASYS, HYDRASHIFT IF assay on the HYDRASYS2 system (Sebia) using HYDRASHIFT 2/4 daratumumab kit or HYDRASHIFT 2/4 isatuximab kit; IMWG, International Myeloma Working Group; Isa, isatuximab; M, male; PD, progressive disease; PR, partial response; sCR, stringent complete response; SD, stable disease; VGPR, very good partial response.

^a^ Creatinine clearance was calculated by Cockcroft‐Gault formula.

^b^ IgG‐κ originating from anti‐CD38 monoclonal antibodies, daratumumab or isatuximab, are italicized.

^c^ IgA‐alone: Epalyzer did not detect the kappa of IgA‐κ so they were judged as IgA‐alone.

The HYDRASHIFT Isa IF assay also completely removed the isatuximab‐based IgG‐κ band which was detected by Epalyzer (IgG‐κ italicized in Table 1) from the gamma region in two of five patients (40%), confirming CR on their response‐based evaluation (patients 13 and 16 in Table 1). The other three patients showed endogenous M‐proteins, IgG‐κ, IgA‐κ, IgG‐λ, and Free‐κ that originated from MM (patients 14, 15, and 17 in Table 1).

### 3.2 Discrepancies in the M‐protein detection between IFE systems

**FIGURE 1:**
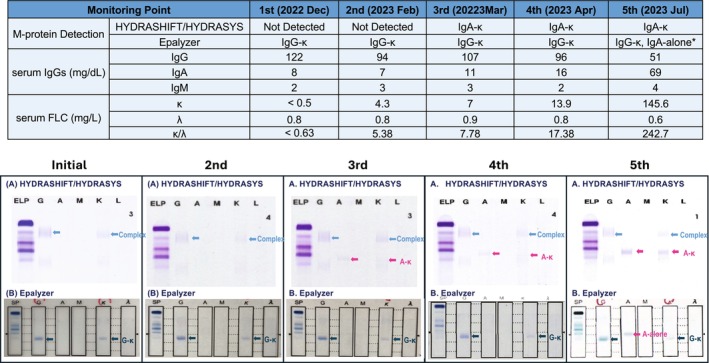
Changes in M‐proteins by each detection method during the course of treatment in Patient 16. (A) HYDRASHIFT/HYDRASYS: The IgG‐κ originating from isatuximab was shifted to the α‐region as the immune complex (blue arrows), so no endogenous M‐protein was detected in the γ‐region at the initial and the second timepoints. At the third timepoint, the IgA‐κ type endogenous M‐protein from the patient relapse continued to be detected (red arrows) as the concentration of IgA slightly increased to 11 mg/dL. (B) Epalyzer: The IgG‐κ originating from isatuximab (dark blue arrows) was detected as native M‐protein, and the system did not detect any IgA until the fifth timepoint, an endogenous IgA without corresponding light chain was detected at the fifth timepoint when the concentration of IgA had increased to 69 mg/dL. Red arrows: endogenous M‐protein originating from Patient, Blue arrows: IgG‐κ immune complex originating from Isatuximab shifted, Dark arrows: IgG‐κ originating from Isatuximab detected as native M‐protein.

Some discrepancies were found with the M‐protein detection between the HYDRASHIFT assay (HYDRASHIFT Dara IF assay or HYDRASHIFT Isa IF assay) on the HYDRASYS2 system (HYDRASHIFT/HYDRASYS) and the IFE assay on the Epalyzer system (Epalyzer). The IFE using Epalyzer could neither avoid the interferences of IgG‐κ originating from the daratumumab/isatuximab treatments, nor detect some types of M‐proteins. One discrepancy was that the kappa aspect of three IgA‐κ type M‐proteins were not detected by Epalyzer, and the sample was judged to be an IgA alone (patients 11 and 15 as shown in Table 1 and Figure S1), and at the fifth monitoring point of Patient 16 in Figure 1. Furthermore, the Free‐κ type M‐proteins of two patients (patients 1 and 15) were not detected by Epalyzer. The discrepancy was especially profound in Patient 15 who had a serious renal impairment and high involved serum FLC, κ:1585 mg/L and λ:6.9 mg/L. The two Free‐κ type M‐proteins, monomer and polymerized, detected by HYDRASHIFT/HYDRASYS in patient 15 (Figure S1), were consistent with the clinical presentation.

### 3.3 Differences in sensitivity and specificity between IFE systems

A small IgA‐κ M‐protein (red arrows in Figure 1) was detected in patient 16 at the 3rd timepoint during monitoring by HYDRASHIFT/HYDRASYS when the patient had relapsed and the concentration of IgA slightly increased from 8 to 11 mg/dL. This was not the case with the Epalyzer. The Epalyzer only detected the IgG‐κ originating from isatuximab as M‐protein (blue arrows) at the third timepoint. When the concentration of IgA increased to 69 mg/dL at the fifth timepoint, the Epalyzer detected an IgA without the corresponding kappa (IgA‐alone) and the IgG‐κ originating from isatuximab (in the right bottom of Figure 1).

## 4 DISCUSSION

In the clinical practice of MM in patients taking isatuximab or daratumumab, the use of HYDRASHIFT/HYDRASYS performed well regarding M‐protein interpretation and accurate CR assessment. These studies suggest that “CR” assessments of anti‐CD38 mAb‐treated patients will increase if they are monitored by HYDRASHIFT/HYDRASYS.

According to the updated results of the IKEMA study using the HYDRASHIFT Isa IF assay,^6^, ^7^ the rate of CR or sCR increased from 40% to 44.1% and the rate of minimal residual disease negative‐CR increased from 20% to 26.3%. The HYDRASHIFT Isa IF assay was used on banked sera to measure endogenous M‐protein in samples suspected of isatuximab interference at the updated results, since the HYDRASHIFT Isa IF assay was not initially available for the IKEMA study. Our cohort size (12 patients with Dara and 5 patients with Isa) is too small to precisely estimate how many patients would be impacted in a larger study, but the results suggested that if the HYDRASHIFT Isa IF assay was utilized on all samples, it would be more effective for accurate CR assessment and the CR/sCR group would be increased.

Furthermore, we found many discrepancies in the IF results between the HYDRASHIFT/HYDRASYS and the Epalyzer. The HYDRASHIFT/HYDRASYS showed higher sensitivity and specificity for M‐protein detection, correctly identifying serum Free‐κ and a low‐level M‐protein (IgA‐κ) which the Epalyzer did not detect. According to Package Inserts, the IFE using HYDRASYS can detect IgG, IgA, IgM, and light chain κ and λ M‐proteins down to 25 mg/dL^12^ and the IFE using the Epalyzer can detect M‐proteins IgG and IgA down to 50 mg/dL, IgM down to 110 mg/dL, and no value is given for light chains.^13^ On this study, the Epalyzer did not detect Light Chains for four samples, no. 1, 11, 12, and 15, with a discrepancy ratio between Hydrasys and Epalyzer of 23.5% (4/17 samples). In our hands, we confirmed using our samples that the HYDRASHIFT/HYDRASYS could detect down to 11.0 mg/dL of IgA‐κ and 12.5–25 mg/dL of IgG‐κ and IgG‐λ, while the Epalyzer could only detect IgG‐κ and IgG‐λ down to 50 mg/dL. These results suggest that M‐protein interpretation and the CR assessments of patients would differ depending on the IFE system used. The results obtained differ depending on the system and reagents used. These differences would be less profound if we used samples from a newly diagnosed cohort, as, among the positive samples, there would be a significant number having high M‐protein concentrations. However, detecting lower M‐protein should be required on the IFE system for the cohort of monitoring patients, and especially those under daratumumab/isatuximab treatment.

These results call attention to which IFE system we should use, not only in response evaluation but also while monitoring patients. We should realize that the Epalyzer may not accurately detect FLCs in patients with renal impairment and may not detect small M‐proteins in relapsed patients.

This has a profound impact on the diagnosis and treatment of MM because IFE is a reference test for M‐protein interpretation and for judging the therapeutic response of patients. Because MM is a serious disease that progresses through repeated relapses and remissions, such risks should not be allowed. However, IFE using the Epalyzer is still popularly used in some countries with no awareness of these serious risks related to medical treatment.

## 5 CONCLUSION

We found that the detection of M‐protein dramatically differs depending on the IFE system used. In the clinical practice of MM in patients being treated with daratumumab/isatuximab, the use of the HYDRASHIFT/HYDRASYS showed good performance, exhibiting higher sensitivity and specificity, providing accurate CR assessment, correctly identifying serum free‐κ in a patient with renal impairment, and detecting small M‐proteins upon relapse. The differences between sensitivity and specificity will strongly influence the response evaluation and monitoring of patients.

## AUTHOR CONTRIBUTIONS

**Yuko Shirouchi:** Data curation (equal); formal analysis (equal); writing – review and editing (equal). **Kazumi Kaihara:** Data curation (equal); writing – review and editing (equal). **Tsunaki Sekita:** Writing – review and editing (equal). **Naoko Amano:** Conceptualization (equal); formal analysis (equal); writing – original draft (equal); writing – review and editing (equal). **Konosuke Nakayama:** Writing – review and editing (equal). **Kazunori Miyake:** Writing – review and editing (equal). **Hitoshi Abe:** Writing – review and editing (equal). **Dai Maruyama:** Conceptualization (equal); data curation (equal); formal analysis (equal); writing – original draft (equal); writing – review and editing (equal). **Hirotoshi Oinuma:** Project administration (equal); writing – review and editing (equal).

## FUNDING INFORMATION

DM received research funding (Ono, Janssen, Eisai, Chugai, Kyowa Kirin, MSD, Zenyaku, Sanofi, Symbio, Takeda, AbbVie, AstraZeneca, BMS, Genmab, Novartis, Otsuka, Taiho, Pfizer, Astellas) and honoraria (Ono, Nippon Shinyaku, Janssen, Mundipharma, Eisai, Chugai, Kyowa Kirin, MSD, Zenyaku, Sanofi, Symbio, Takeda, AbbVie, AstraZeneca, BMS, Genmab, Novartis), YS received honoraria (Janssen, Sanofi), and all the other authors have nothing to disclose.

## ETHICS STATEMENT

The study design was approved by the institutional review board at the Japanese Foundation for Cancer Research. The requirement for written informed consent was waived by the board, and patients were offered an opt‐out option.

## Supporting information


Figure S1.

## Data Availability

Individual participant data will not be shared, because informed consent was not obtained for data sharing; the protocol will also not be shared due to the institutional policy.
