# Supplementary material for: Impact of M‐protein detection on the response evaluations of patients undergoing treatment with the IgG‐κ monoclonal antibodies daratumumab or isatuximab, and discrepancies between immunofixation electrophoresis (IFE) systems and reagents
Source: Cancer Med. 2024 Aug 23;13(16):e70128. doi: 10.1002/cam4.70128 (PMC11342076; doi:10.1002/cam4.70128)
Supplement: Supplementary file 1 — Figure S1. [file CAM4-13-e70128-s001.docx]

A. IFE using HYDRASHIFT/HYDRASYS B. IFE using Epalyzer


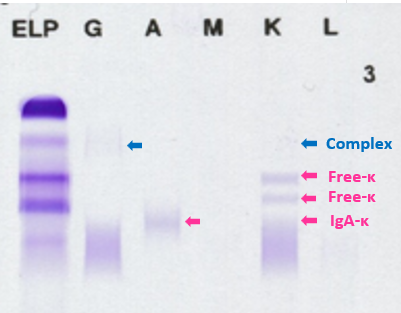


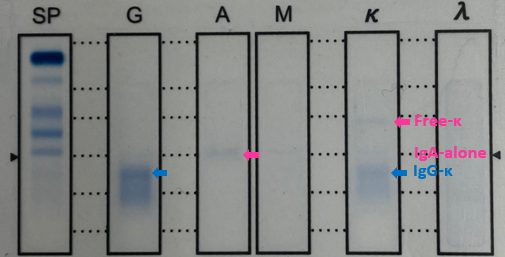


Red arrows: Endogenous M-proteins originating from Patient, Blue arrows: the IgG-κ originating from Isatuximab judged as native M-protein.

Red arrows: Endogenous M-proteins originating from Patient, Blue arrows: the IgG-κ immune complex originating from Isatuximab.

**Figure S1. Comparison between two IFE systems in Patient 15**

The IFEs, electrophoresis of serum subsequently immunofixed with anti-IgG, IgA, IgM, κ, and λ, were performed on the HYDRASYS2 system (Sebia) using HYDRASHIFT 2/4 Isatuximab kit on the left (A), and on the Epalyzer2 system (Helena) on the right (B).

Red arrows: bands judged to be endogenous M-proteins. Blue arrows: bands indicate the IgG-κ originating from Isatuximab.

A: The IgG-κ immune complex originating from isatuximab was shifted (blue arrows) and the type of endogenous M-protein was judged to be IgA-κ and two Free-κ (red arrows), indicating single and polymerized Free-κ, which is consistent with severe renal impairment and a high level of serum FLC, 1585 mg/L.

B: Isatuximab was detected as an IgG-κ type M-protein (blue arrows). An endogenous IgA band was detected without corresponding kappa, and only one Free-κ band was detected (red arrows).
